# *Ganoderma lucidum*-Mediated Green Synthesis of Silver Nanoparticles with Antimicrobial Activity

**DOI:** 10.3390/ma16124261

**Published:** 2023-06-08

**Authors:** Mariana Constantin, Iuliana Răut, Raluca Suica-Bunghez, Cristina Firinca, Nicoleta Radu, Ana-Maria Gurban, Silviu Preda, Elvira Alexandrescu, Mihaela Doni, Luiza Jecu

**Affiliations:** 1 National Institute for Research & Development in Chemistry and Petrochemistry-ICECHIM, 202 Independentei Spl., 060021 Bucharest, Romania; marriconstantin@yahoo.com (M.C.); iulia_rt@yahoo.com (I.R.); raluca_bunghez@yahoo.com (R.S.-B.); firincacristina@yahoo.com (C.F.); nicolbiotec@yahoo.com (N.R.); elviraalexandrescu@yahoo.com (E.A.); mihaela.doni@icechim.ro (M.D.); 2 Faculty of Pharmacy, Titu Maiorescu University, 16 Bd. Gh. Sincai, 040441 Bucharest, Romania; 3 Faculty of Biology, University of Bucharest, Splaiul Independentei 91-95, 050095 Bucharest, Romania; 4 Faculty of Biotechnology, University of Agronomic Sciences and Veterinary Medicine of Bucharest, 59 Mărăşti Boulevard, 011464 Bucharest, Romania; 5 Institute of Physical Chemistry “Ilie Murgulescu”, 202 Splaiul Independentei Spl., 060021 Bucharest, Romania; predas01@yahoo.co.uk

**Keywords:** *Ganoderma lucidum*, green synthesis, silver nanoparticles, antimicrobial activity

## Abstract

“Green chemistry” is a simple and easily reproductible method that provides nanoparticles characterized by better stability and good dispersion in an aqueous solution. Nanoparticles can be synthesized by algae, bacteria, fungi, and plant extracts. *Ganoderma lucidum* is a commonly used medicinal mushroom with distinctive biological properties, such as antibacterial, antifungal, antioxidant, anti-inflammatory, anticancer, etc. In this study, aqueous mycelial extracts of *Ganoderma lucidum* were used to reduce AgNO_3_ to form silver nanoparticles (AgNPs). The biosynthesized nanoparticles were characterized by UV-visible spectroscopy, scanning electron microscopy (SEM), X-ray diffraction (XRD), and Fourier transform infrared spectroscopy (FTIR) analysis. The maximum UV absorption was obtained at 420 nm, which represents the specific surface plasmon resonance band for biosynthesized silver nanoparticles. SEM images showed particles as predominantly spherical, while FTIR spectroscopic studies illustrated the presence of functional groups that can support the reducing of ion Ag^+^ to Ag(0). XRD peaks ratified the presence of AgNPs. The antimicrobial effectiveness of synthesized nanoparticles was tested against *Gram*-positive and *Gram*-negative bacterial and yeasts strains. The silver nanoparticles were effective against pathogens, inhibiting their proliferation, and thus reducing the risk to the environment and to public health.

## 1. Introduction

The increasing tendency of microbial diseases, together with drug-resistance to antibiotics, has become major a public health concern, determining the search for alternative treatments through development of nanotechnology. Nanotechnology offers viable solutions in many applications, and among them, metallic nanoparticles are known for their potent antibacterial activity against human pathogens, providing the most effective results [1,2,3].

The so called “green chemistry” synthesis of nanoparticles by biological methods has many advantages over conventional chemical and physical methods, such as being safe and nontoxic for the environment and reducing energy and resource consumption. These methods are simple and easily reproductible, offering nanoparticles characterized by better stability and good dispersion in an aqueous solution.

In the last decade, many review articles have been dedicated to green synthesis of silver nanoparticles, with emphasis on the synthesis mechanism on a cellular and molecular level as well as on their extended applications [2,3,4,5,6,7,8,9]. Silver nanoparticles (AgNPs) have been studied intensely due to their physical and chemical properties (optical, electrical, thermal, and biological) useful in many fields, such as medicine, health, food, and industry. Accordingly, the application of AgNPs in the biomedical domain has registered particular attention due to their effectiveness in dental applications, bone healing, bone cement, impregnating or coating of catheters, and wound healing [10]. Meanwhile, AgNPs have been proven to provide efficient protection for plants against bacteria, fungi, as well as against pests that produce enormous damage to agricultural crops annually [11,12,13]. Regarding the industrial applications, AgNPs can be useful to maintain food quality via regulation of foodborne pathogens [6,14], and also have excellent commercial value for cosmetics, showing antioxidant properties [15,16].

Nanoparticles can be synthesized by algae, bacteria, fungi, and plant extracts. The biogenic formation of silver nanoparticles has been achieved with many bacterial species, such as *Bacillus cereus* [17], *Bacillus subtilis* [18], *Rhodococcus roppenstedtii* [19], and a consortium of *Rhodococcus, Brevundimonas*, and *Bacillus* [20]. Yeast strains are also mentioned in the synthesis of metallic nanoparticles, namely those belonging to *Candida* and Schizosaccharomyces genera [21].

Many fungi are reported in the synthesis of silver nanoparticles, and among these, the most studied are *Fusarium* [22], *Penicillium* [23], endophyte *Talaromyces sp.* [24], and various *Aspergillus* species [25,26,27]. Fungi are preferred for biogenic synthesis due to their high tolerance of metals, simple conditions for cultivation, good biomass production, and to its ability to secrete large quantities of extracellular proteins that are involved in biosynthesis. Among species reported to be good candidates for the synthesis of nanoparticles, *Basidiomycetes* have also been mentioned, which are a group of macro fungi secreting extracellular enzymatic systems, proteins, polysaccharides, and secondary metabolites with diverse functions. The species studied belong to *Pleurotus, Lentinus, Grifola*, and *Ganoderma* genera, with the properties of biogenic nanoparticles being closely related to the mushroom species used. [6,12,28,29,30,31,32].

A major interest is to find evidence of the capacity of certain microorganisms, especially fungal strains, to synthesize nanoparticles in mild conditions with low costs. Furthermore, depending on the fungus used, specific parameters of fungal growth as well as the time required for actual biosynthesis could be decisive in the process and in further utilization of AgNPs. In this regard, our attention was focused on *Ganoderma lucidum,* a commonly used medicinal *Basidiomycete* with distinctive biological properties, such as antibacterial, antifungal, antioxidant, anti-inflammatory, anticancer, etc. In addition, the *Ganoderma* species biosynthesize silver and gold nanoparticles, an important feature for their use in medical applications where the nanoparticles must be compatible and obtained by non-toxic methods. AgNPs are synthesized by *Ganoderma* through an extra or intracellular mechanism, depending on the location where nanoparticles are synthesized. The characteristics of nanoparticles, namely size, morphological shape, and surface characteristics, influence their properties, playing a significant role in subsequent use.

In the present work, reflecting on the above-mentioned aspects, an attempt has been made to develop a low-cost, simple, and eco-friendly method for the microbial synthesis of AgNPs with antimicrobial activities. The nanoparticles were synthesized using mycelium extract from *Ganoderma lucidum,* and were characterized by analysis via UV-visible spectroscopy, FTIR spectra, XRD, and SEM images. The antimicrobial activity of nanoparticles carried out using a diffusion method was evaluated against three bacteria and, respectively, two yeasts strains. This study reveals that the biosynthesized silver nanoparticles expressed good antimicrobial activity against pathogenic microorganisms posing health hazards to human beings.

## 2. Materials and Methods

### 2.1. Cultivation of Ganoderma lucidum

From a newly prepared stock culture (about 7 days incubation at 30 °C), actively growing mycelia (1 piece of 5 mm diameter) were grown for 10 days in absence of light on agar solid medium with malt extract. The medium composition was (g/L) 30, malt extract (Sigma-Aldrich, Merck KGaA, Darmstadt, Germany); 3, peptone (Scharlau, Scharlab S.L., Spain); 20, agar (Scharlau, Scharlab S.L. Spain); pH = 5.6 ± 0.2 at 25 °C. Pieces of fungal mycelium were inoculated in liquid potato dextrose broth (PDB, Scharlau, Scharlab S.L., Spain), which had the following composition (g/L): 4, potato peptone; 20, glucose; pH = 5.6 ± 0.2 at 25 °C. The flasks were incubated for 7 days, in static conditions and in darkness. The mycelium from the culture broth was washed 3–4 times with sterile water to remove the remaining components of the culture medium. After this, 10 g of wet mycelium was suspended in 100 mL of sterile water and incubated under agitation at 150 rpm, 30 °C, for 3 days in darkness.

### 2.2. Preparation of AgNPs

AgNPs were prepared by using the green synthesis approach, as follows: the fungal aqueous extract obtained in the above subsection was filtered on Whatman filter paper No. 1, and 40 mL of filtrate was mixed with the metallic precursor, as 10 mL of silver nitrate solution. Stock solution of silver nitrate (Carlo Erba Reagents GmbH, Germany) was prepared by dissolving 4.24675 g of silver nitrate in 250 mL of distilled water (0.1 mol/L). The mixture of aqueous mycelium extract and nitrate solution was observed for 7 days to detect the changing of colour due to the appearance of AgNPs.

### 2.3. UV–VIS Spectroscopy Study

The UV-Vis spectra of samples were recorded in the wavelength range of 250 to 650 nm, at room temperature, utilizing the UV-VIS Cintra 202 spectrophotometer, in 10 mm standard quartz cuvettes, by using ultrapure water as a blank. The nanoparticle samples were placed in a sample compartment and recorded at different time intervals. The UV-Vis spectrophotometer was operated at 220 V, at a temperature of 20 ± 2 °C and 60–70% humidity [28,30].

### 2.4. Fourier Transform Infrared Spectroscopy (FTIR) Analysis

The spectral characterization of the biosynthesized AgNPs was recorded by Fourier transform infrared spectroscopy (Perkin Elmer FTIR) using the dried powder of the synthesized AgNPs, in a spectral range of 600–4000 cm^−1^ at room temperature [28,30].

### 2.5. XRD Analysis

X-ray diffraction was used to evaluate the crystalline structures in the gel sample [29,30]. The measurements were performed with an Ultima IV diffractometer (Rigaku Corp., Tokyo, Japan) equipped with parallel beam optics, using CuKα radiation (λ = 1.5418 Å), at 40 kV and 30 mA, over the 5–80° range, at a scanning rate of 1°·min^−1^, with a step width of 0.02°, in continuous mode, at room temperature and atmospheric pressure. The phase identification was performed using the PDXL’s proprietary Hybrid Search/Match algorithm, where PDXL is integrated X-ray powder diffraction software from Rigaku Corporation, connected to the International Centre for Diffraction Data, Powder Diffraction File database (ICDD PDF-2).

### 2.6. Scanning Electron Microscopy (SEM)

The surface morphology and structure of the nanoparticles were evaluated using a FEI Quanta 200 scanning electron microscope, using a low vacuum mode (133 Pa chamber pressure) and an acceleration voltage of 30 KV. To protect the less conductive areas, the specimens were covered by a 5 nm layer of gold, using a Quorum 150 R ES Plus Sputter Coater.

### 2.7. Size Determination of Silver Nanoparticles

A Zetasizer Nano ZS ZEN 3600 (Malvern) instrument was used to measure the average particle diameter of the dispersion of silver nanoparticles. A dispersion of 1 mL sample in 2 mL distilled water was sonicated for 20 min in an ultrasonication bath. The DLS measurement of the silver nanoparticle dispersion revealed monomodal size distributions.

### 2.8. Antimicrobial Activity

The green-synthesized AgNPs were further investigated via the common agar diffusion method for antimicrobial activity against several pathogens, such as *Staphylococcus aureus* ATTC 25923, *Escherichia coli* ATTC 25922, *Pseudomonas aeruginosa* ATTC 27853, *Candida albicans* ATTC 10231, and *Candida parapsilosis* ATTC 22019. The bacterial strains were cultured on the Mueller–Hinton medium with the following composition (g/L): 17.5, Scharlau dehydrated powder; 1.5, peptone; 2, meat infusion solids; 15, agar. The two *Candida* strains were cultured on Sabouraud medium (g/L): 10, Scharlau dehydrated powder; 40, glucose; 15, agar. Both culture media were prepared, and after autoclaving, they were cooled and poured onto Petri plates. The pathogen cultures were swabbed onto sterilized agar media with a sterile cotton swab. The control was considered the aqueous extract of *Ganoderma* mycelium. With a sterile micro pipette, two doses of 30 µL and 50 µL of aqueous mycelium extract (as control) and AgNP suspension, respectively, were applied to agar medium inoculated with pathogens. After that, the plates were incubated at 37 °C (bacteria) and 28 °C (yeasts) for 24 h. At the end of the incubation period, the zones of inhibition were observed and the diameters were measured in mm. Each assay was carried out in triplicate and the mean and standard error was calculated based on triplicate tests. The inhibition of pathogen growth by a specific antibiotic impregnated in a paper disk represented a positive test. Related to each microorganism, the following antibiotics were used: *S. aureus*, 2 µg clindamycin; *E. coli*, 5 µg ciprofloxacin; *P. aeruginosa*, 10 µg gentamicin; *C. albicans* and *C. parapsilosis*, 25 µg fluconazole [33].

## 3. Results

### 3.1. Synthesis of Silver Nanoparticles

**Figure 1:**
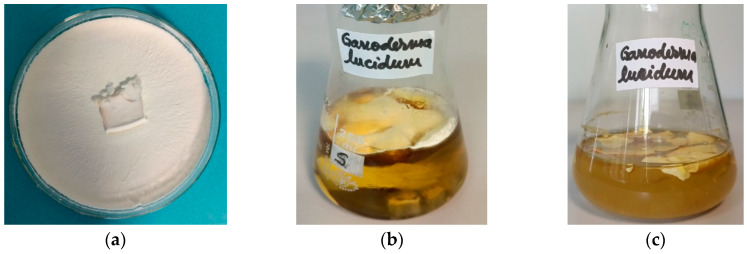
Images of *Ganoderma lucidum* cultures. (**a**) Pure culture of *Ganoderma lucidum* in a malt extract medium. (**b**) *Ganoderma lucidum* culture in a liquid PDB medium, after seven days of incubation in darkness in static conditions. (**c**) Aqueous extraction in sterile distilled water of fungal mycelium.

The silver nanoparticles were synthesized according to the experimental protocol presented. The mycelium of *Ganoderma lucidum* was inoculated in potato dextrose broth liquid medium. The steps of the process are presented in Figure 1, including the cultivation of *Ganoderma* in a solid medium (Figure 1a) and then in a liquid medium (Figure 1b), and, finally, the water extraction of fungal mycelium (Figure 1c) as a common and easy way to obtain major bioactive components.

### 3.2. Visual Observation of Colour Change during Green Synthesis

**Figure 2:**
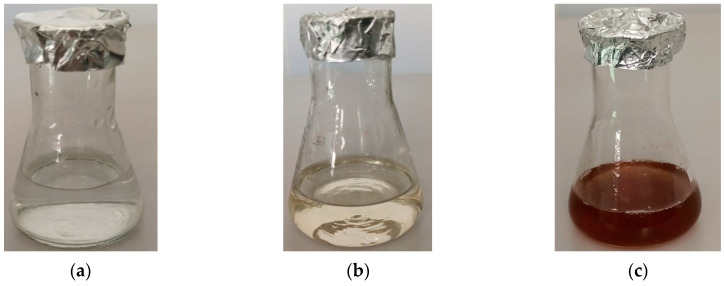
Visible observation of green synthesis of silver nanoparticles by *Ganoderma lucidum.* ****(**a**) Erlenmeyer flask with AgNO3 precursor solution; (**b**) Erlenmeyer flask with aqueous extract of mycelium from *Ganoderma lucidum.* (**c**) Erlenmeyer flask with suspension of AgNPs after three days of contact between the AgNO_3_ solution as metal precursor and aqueous extract of mycelium from *Ganoderma lucidum*.

The synthesis of nanoparticles was evidenced by the change in colour from transparent to yellowish brown in a solution comprising silver salt and aqueous mycelium extract. This colour change reflects a primary indication of AgNP synthesis occurring in the presence of mycelium extract. As can be seen, the colour remained unchanged in the flask containing the aqueous mycelium extract (Figure 2). The aqueous extract sustained the colour even after 170 h, indicating that AgNPs were dispersed in the solution.

### 3.3. UV-Vis Spectra

**Figure 3:**
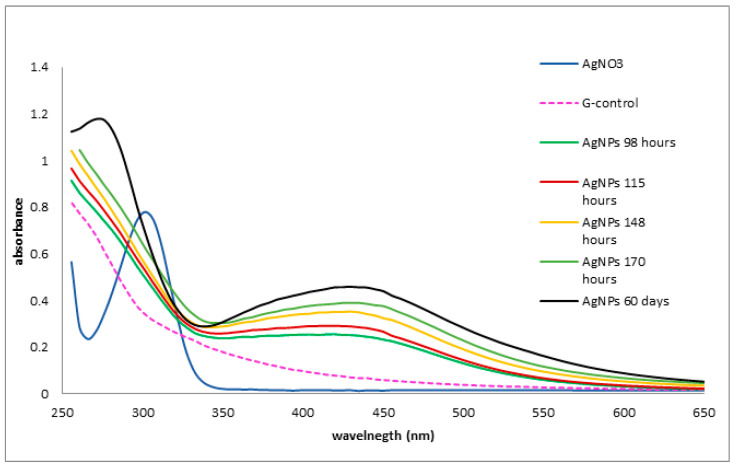
The ultraviolet-visible spectra of biosynthesized silver nanoparticles. The spectra were registered at different time intervals, even after 60 days.

The formation of AgNPs was confirmed by the results of UV-Vis spectra, in the range of 250–650 nm. As shown in Figure 3, the specific SPR (surface plasmon resonance) band for biosynthesized AgNPs at different time intervals was found at 420 nm. The value of the absorption peak at 420 nm indicates the constancy of synthesized AgNPs. As can be seen, the specific plasmon band is not symmetrical, due to the presence of aggregate particles. The influence of time on nanoparticle synthesis was evidenced by the measurements at different times. The absorbance at 420 nm increased with the incubation time of the mixture containing silver nitrate and aqueous mycelium extract.

### 3.4. FTIR Results

**Figure 4:**
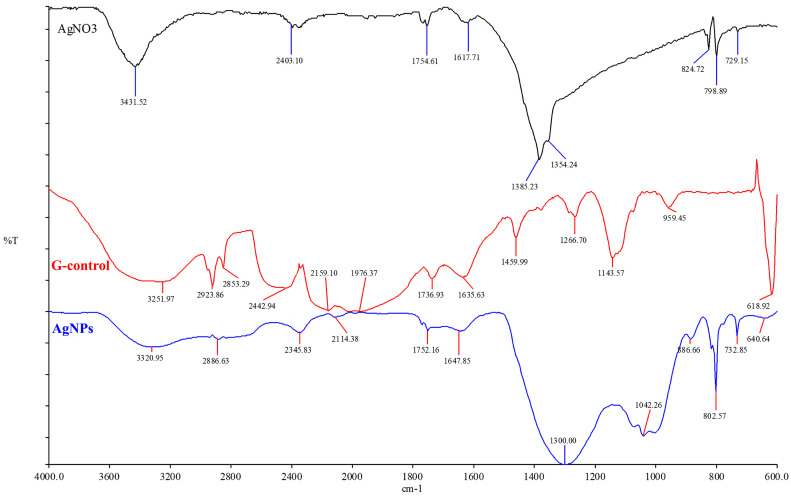
ATR Fourier transform infrared spectroscopy analysis of biosynthesized silver nanoparticles and aqueous extract of mycelium from *Ganoderma lucidum*.

FTIR spectroscopy is a valuable tool to investigate the mycogenic synthesis of AgNPs, providing relevant information about the chemical bonds and molecular structures responsible for the bio-reduction of Ag^+^ and capping/stabilization of AgNPs. Figure 4 depicts the FTIR spectra of the mycelium extract (G-Control), nanoparticles synthesized by G. lucidum (AgNPs), and silver precursor (AgNO_3_). The functional groups of the synthesized AgNPs were identified based on the FTIR analysis. The major peaks of the silver nitrate sample (AgNO_3_) were observed at 3431.52, 2403.10, 1754.61, 1617.71, 1385.23, 1354.24, 824.72, and 798.89 cm^−1^. In the vast majority, a slight shift to lower values of the corresponding peaks were observed, in the biosynthesized nanoparticles (AgNPs) sample, at 3320.95, 2345.83, 1752.16, 1300.00, 802.57, and 732.85 cm^−1^.

In the aqueous mycelium extract (G-Control), the following bands were identified: the broadband at 3251.97 cm^−1^ corresponds to -OH stretching from alcohol and phenolic compounds; the peak at 2923.86 cm^−1^ indicates the C-H stretching of alkanes; the peak at 1736,93 cm^−1^ is assigned to carbonyl C=O stretching of saturated aliphatic esters; the peak at 1635.63 cm^−1^ could be denoted by carbonyl stretching vibration in amide I proteins; the peak at 1378.91 cm^−1^ indicates symmetric bending of aliphatic CH and triterpene compounds; the peak at 1143.57 could be assigned to triterpene compounds (C-O); and the peak at 618.92 cm^−1^ could be assigned to CH_2_ groups.

Moreover, it can be observed that the domain between 1459.99 and 1266.70 cm^−1^, assigned to the variation in amide III, corresponds mainly to proteins. The variation in carbohydrate content is recorded at 900–960 cm^−1^. In the spectra of biosynthesized nanoparticles, AgNPs, the intensity of the peak at 1635.63 cm^−1^ from the mycelium extract (G-Control) shifted at 1647.85 cm^−1^ and become wider. This aspect could be explained by the assumption that some carbonyl groups were oxidized to carboxylic ester groups, which were involved in the reducing of ion Ag^+^ to Ag(0) [34]. We can conclude that the nanoparticles were surrounded by proteins and metabolites that contain hydroxyl groups. The residues of amino acids can function as capping agents, stabilizing the biosynthesized nanoparticles.

### 3.5. XRD Analysis

**Figure 5:**
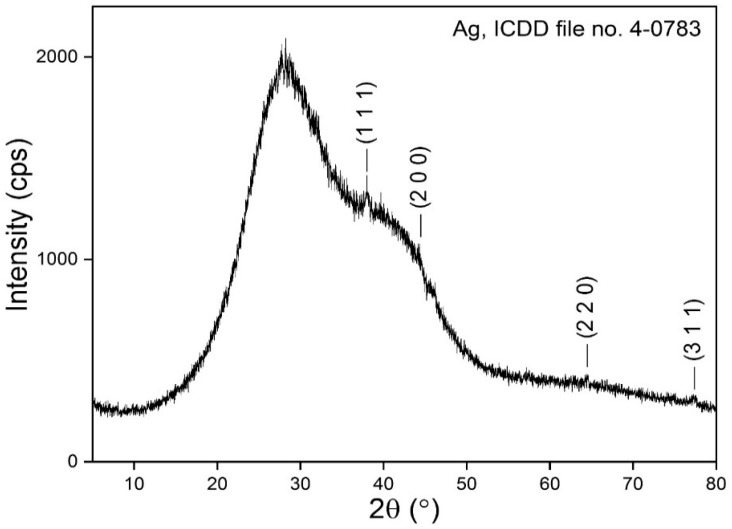
XRD pattern of extracellular synthesized silver nanoparticles using *Ganoderma lucidum*.

The diffracted intensities were recorded from 20° to 80°, with Figure 5 showing the X-ray pattern of the gel sample. X-ray diffraction revealed the presence of crystalline and amorphous phases. Low-intensity diffraction lines, located at 2θ angles 37.93°, 44.29°, 64.49°, and 77.31°, matched well against ICDD file no. 4-0783, corresponding to Ag, Silver-3C. The peak corresponding to plane (111) is more intense, indicating that this direction of particle growth is major. The obtained Bragg reflections of AgNPs located at 2θ angles are very similar to those reported by other studies [12,29,30,35].

### 3.6. SEM Results

**Figure 6:**
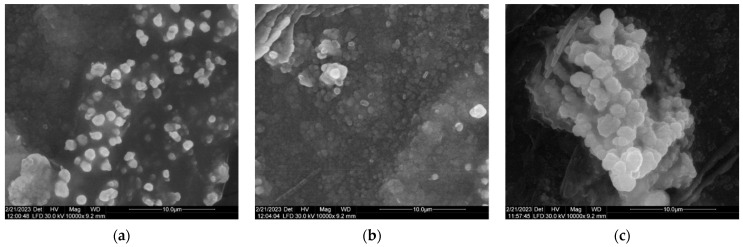
SEM images of AgNPs showing their morphology (10.000X). (**a**) The particles having a predominantly spherical shape appear individually scattered. (**b**) Agglomeration of particles which are not aggregated. (**c**) Particle aggregation in indefinite form.

The SEM images of AgNPs in Figure 6 provided data about the morphology of silver biosynthesized nanoparticles. The particles are predominantly spherical, but the aggregation of particles can be observed.

The agglomeration of the nanoparticles observed in Figure 6b can be attributed probably to the presence of biomolecules from the culture medium of *Ganoderma lucidum*, as well as to the metabolites which are responsible for biosynthesis.

### 3.7. Dynamic Light Scattering (DLS) Analysis

**Figure 7:**
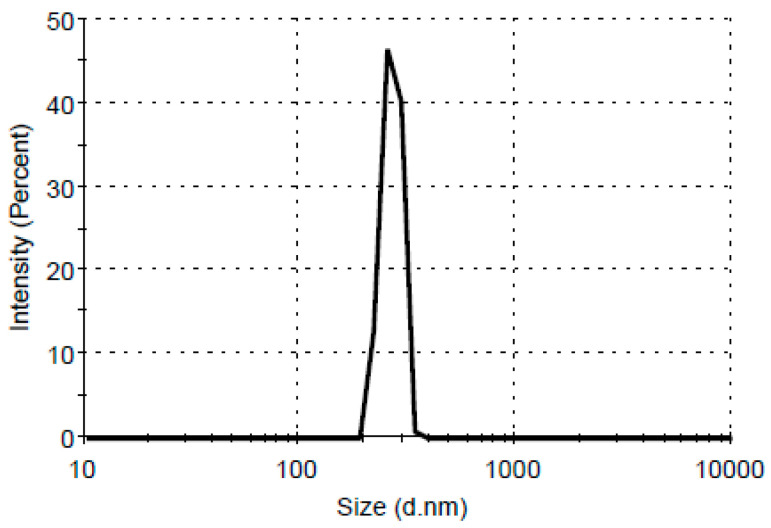
DLS size distribution by intensity of silver nanoparticles synthesized by mycelium extract from *Ganoderma lucidum*.

The size and distribution of nanoparticles are significant to quality control of nanoparticle synthesis. The capacity of nanoparticles to diffuse through cell membranes is connected to their size and is relevant to antimicrobial activity. The results of DLS analysis are presented in Figure 7. In the graphical representation of particle size distribution as function of the intensity, a single peak can be seen for nanoparticles of a certain diameter.

### 3.8. Antimicrobial Activity

**Figure 8:**
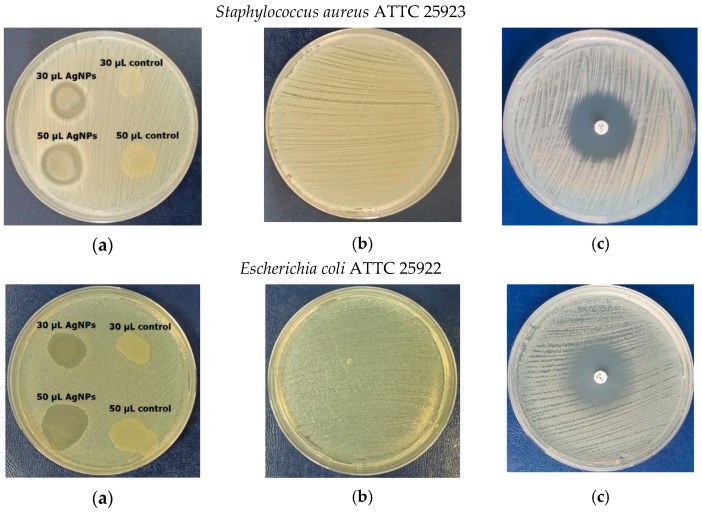

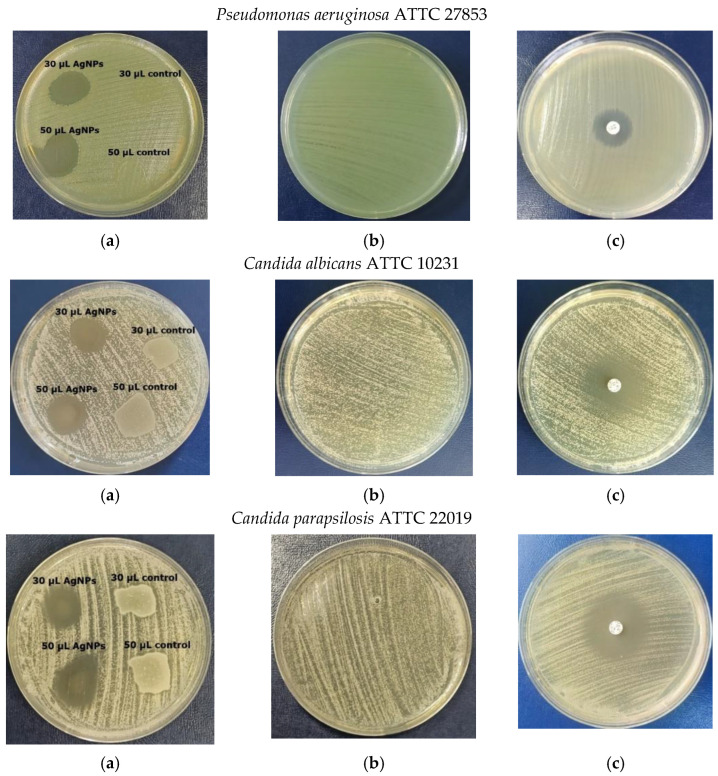
Antimicrobial activity of AgNPs versus pathogens. (**a**) Zones of pathogen inhibition in Mueller–Hinton and Sabouraud plates for bacteria and yeasts, respectively, produced by control and AgNPs suspension (30 and 50 µL doses). (**b**) Pure culture of pathogen in agar medium. (**c**) Positive test—inhibition of pathogen growth by specific antibiotic impregnated in a paper disk. The control was considered the aqueous extract of *Ganoderma* mycelium.

**Figure 9:**
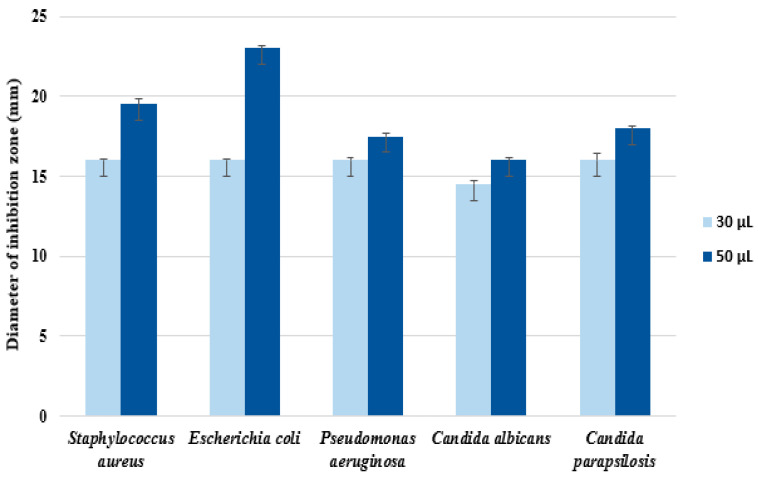
Antimicrobial activity of AgNPs (doses of 30 and 50 µL, respectively) versus pathogens. Data represent the mean ± standard error of triplicate samples from three identical experiments performed in the same experimental conditions.

The green-synthesized AgNPs were further investigated for antimicrobial activity against two Gram-negative bacteria, *Escherichia coli* and *Pseudomonas aeruginosa*, one Gram-positive strain, *Staphylococcus aureus*, and two yeasts, *Candida albicans* and *Candida parapsilosis*. Tests related to the susceptibility of selected microbial pathogenic strains versus biosynthesized silver nanoparticles were performed on Petri plates in agar medium using the diffusion method (Figure 8). The nanoparticles as antimicrobial agents diffused into the agar and inhibited germination and growth of the test microorganisms. The diameters of inhibition growth zones reflected the susceptibility of pathogens versus the antimicrobial agents (Figure 9).

In experimental tests with a dose of 30 μL from the AgNP solution, no significant difference in antimicrobial efficiency, expressed as a diameter of the inhibition zone for each pathogen, was observed. The registered diameter values were in the range of 14.8–16.0 mm, with the maximum for *Pseudomonas aeruginosa* (16.1 ± 0.2 mm), followed by *S. aureus* and *C. parapsilosis* (equal value of 16.0 ± 0.1 mm), *E. coli* (15.9 ± 0.1 mm), and the lowest value of 14.5 ± 0.2 mm for *C. albicans*.

It can be noted that an increasing amount of nanoparticles was correlated with an increase in the efficiency of pathogen inhibition, with a 50 μL dose inducing an increase in diameter of, respectively, 19.0% for *S. aureus*, 29% for *E. coli*, 8.1% for *P. aeruginosa*, 9.1% for *C. albicans,* and 11.3% for C. *parapsilosis*, as compared to those obtained in tests with a 30 μL dose. In this case, the susceptibility of pathogens versus antimicrobial agents, expressed as a diameter of the inhibition zone, decreased in the following order: 23.0 ± 0.2 mm for *E. coli* < 19.5 ± 0.3 mm for *S. aureus* < 18.1 ± 0.1 mm for *C. parapsilosis* < 17.5 ± 0.2 mm for *P. aeruginosa*, and for *C. albicans* < 16.0 ± 0.2 mm. Overall, the antimicrobial effect of AgNPs was more intense against bacteria, and relatively weaker against fungi, with the behaviour towards C. *parapsilosis* being closer to bacteria than the other strain of *Candida*.

## 4. Discussion

The increased interest in fungal-mediated synthesis of nanoparticles compared to bacteria is mainly due to their ability to tolerate high concentrations of metal and metalloids and secrete extracellular proteins acting in the redox process of reducing metal ions to metal. In addition, fungi are easily cultured in submerged or solid-state fermentation processes, producing large quantities of metal nanoparticles, mostly by extracellular pathways [7]. The properties of biogenic nanoparticles depend on the strain and fungal extract used, on the type and concentration of the precursor, and by the conditions of fungal cultivation (e.g. temperature, pH values, and incubation time). These properties determine future applications. Despite the great numbers of studies dedicated to biogenic nanoparticles, there are still aspects to be clarified, such as the particular compounds of the fungal secretome involved [7]. Researchers [7] reported on a comprehensive review of the reduction mechanisms of fungi used in the biosynthesis of various metal nanoparticles (Au, Ag, Pt, Fe, Pd, Cu, Te) and underlined the need to extend research on nanosynthesis processes for the fabrication of particles with desired properties.

Scientists agree that the antimicrobial activity of AgNPs occurs through four mechanisms, as follows: (i) adhesion on the cell wall and membrane surfaces; (ii) damage to the intracellular structure and molecules; (iii) generation of reactive oxygen species and free radicals, thus inducing cytotoxicity; and (iv) interfering with signal transduction pathways [36,37,38].

A recent report mentions that AgNPs are acting in a new antibacterial paradigm, since the antibacterial mechanism of AgNPs effectively avoids the occurrence of bacterial resistance [39]. In a comprehensive review exploring the benefits of NPs synthesized by macro fungi, the authors [6] argued for the need for more studies on the specific mechanism pathways for the biosynthesis of nanoparticles mediated by microorganisms.

*Ganoderma lucidum* is one of the “magic mushrooms”, with a long history of use in traditional medicine. The choice of *Ganoderma lucidum* was attributed to its antimicrobial properties, as well as its capacity to secrete more than 400 bioactive compounds identified in fungal extracts, such as steroids, polysaccharides, alkaloids, triterpenoids, sesquiterpenoids, meroterpenoids, steroids, sterols, nucleotides, fatty acids, vitamins, and minerals. All these compounds can act as reducing and stabilizing agents in the biosynthesis of nanoparticles [12].

In our study, the biosynthesis of AgNPs was evidenced by the appearance of a yellowish-brown colour in a solution containing an aqueous extract of mycelium from *G. lucidum* and a metal precursor salt, AgNO_3_ (Figure 2). The intensity of the reddish-brown colour increased with time because of excitation of surface plasmon resonance (SPR) and reduction of AgNO_3_ [40].

UV-visible spectroscopy is primarily used as a characterization technique for NPs. Generally, according to the literature, the specific UV-Vis absorption peak of AgNPs occurs in the well-documented domain of 380–450 nm, depending on some nanoparticle characteristics, such as size, shape, and particle agglomeration [26,28,41,42]. The silver nanoparticles synthesized through metabolites from *Ganoderma lucidum* presented a maximum absorption at 420 nm. In addition, UV absorbance was recorded after 2 months and the shape, broadness, and specific absorbance at 420 nm remained constant, a sign of nanoparticle stability (Figure 3) [35]. Our results were in agreement with those obtained from a Vietnamese *Ganoderma lucidum* extract, where AgNPs were considered highly stable since the location of the SPR band remained unchanged after 5 months [29].

FTIR spectroscopy is the most commonly used method for the analysis of nanoparticles obtained through biosynthesis. The spectra could provide evidence of the presence of organic molecules acting as capping and stabilizing ligands for NPs. Generally, the migration of the bands in FTIR spectra to lower values indicates the involvement of above mention organic molecules in nanoparticle synthesis and, also the interaction with other functional groups [43]. In FTIR spectra of the mycelium extract, a broadband was found at 3251.97 cm^−1^ corresponding to -OH stretching from alcohol and phenolic compounds. According to Nguyen et al. [31], these functional groups make a major contribution to the reduction of Ag+ to Ag(0) from nanoparticles by transferring electrons. Our FTIR data indicated that the synthesized AgNPs were enclosed by *Ganoderma*-secreted proteins and amino acids, which may be responsible for the stability of the AgNPs (Figure 4). Zhu and Tan [44] reported that FTIR bands of biomolecules contained in aqueous mycelium extracts from *G. lucidum*, namely terpenoids and polysaccharides, are presented in the region of 1150 to 1000 cm^−1^ and 1760 to 1600 cm^−1^, respectively. Considering these aspects, we can conclude that FTIR spectroscopic studies illustrated the presence of functional groups that can form a layer covering the silver nanoparticles to ensure their stabilisation.

The XRD values harmonized well with the cubic crystal lattice planes (111), (200), (220), and (311), respectively, of silver metal. There are several reports that present results similar to our XRD analysis. The crystalline pattern of AgNPs obtained via a biological method using G. applanatum methanol crude extract indicated peaks located at 39.01°, 46.57°, 65.21°, and 78.13° [30]. Ethanol, through hot extraction of mycelium from *G. lucidum*, was used to synthesize AgNPs, and XRD analysis showed three main peaks with their corresponding intensity at planes 100, 220, and 311 [12].

The SEM images (Figure 6) showed a similarity with nanoparticles reported in the literature. Thus, the images are the same in appearance as the particles synthesized by the wild edible mushroom *Pleurotus giganteus* [45]. Silver nanoparticles obtained from *Fusarium oxysporum* were aggregated in a spherical shape [46]. In some cases, the SEM micrographs indicated the aggregation of silver nanoparticles synthesized by *Ganoderma applantum* [30]. The nanoparticles mediated by mushrooms may be monodispersed or polydispersed with different sizes [47].

DLS analysis of silver nanoparticle distribution as a function of intensity depends upon the rate of fluctuation in intensity of the laser beam with respect to particles of different size [28,31] There is an inverse correlation between dimensions of AgNPs and their antibacterial activity. It has been demonstrated that the smaller and medium-sized dimensions of the nanoparticle facilitate the penetration into bacterial cell walls, damaging the internal organelles [38]. Nanosized particles (less 100 nm) are used in drug delivery and in the development of biosensors [15]. AgNPs obtained from aqueous extracts of wild *Ganoderma lucidum* exhibited a particle size distribution between 25 and 150 nm, with an average diameter of 55 nm, suitable as an antimicrobial agent and as a nanosensor for environmental monitoring and remediation [31]. The diameter, defined as a hydrodynamic diameter, is the diameter of a sphere that has the same translational diffusion coefficient as the particles. In the present investigation, the DLS results corroborated with SEM images showed the aggregation of the particles. It is vital to overcome this phenomenon due to attractive forces between nanoparticles.

The silver nanoparticles have been evaluated for their antimicrobial activity against several bacteria and yeasts, all well known as pathogens infesting hospitals and health care environments. Therefore, we have selected a Gram-positive bacteria, *Staphylococcus aureus*, and two Gram-negative bacteria, *Pseudomonas aeruginosa* and *Escherichia coli*, motivated by the large number of pathogens and infestations related to these microorganisms, which are commonly associated with hospital and residential environments. Furthermore, Gram-negative bacteria have a remarkable cell wall structure that confers a strong protection on the whole cell and resistance to commonly used antibiotics. *Pseudomonas aeruginosa* is one of the leading causes of nosocomial infections, expressing antibiotic resistance as a natural trait and characterized by significant survival and persistence in various environments. Pathogenic Escherichia coli are mainly responsible for neonatal meningitis, urinary tract infections, and intestinal diseases. *Staphylococcus aureus*, a facultative anaerobic Gram-positive coccus, is the main cause of human infection in the respiratory tract and on the skin. Candida parapsilosis is one of the most common *Candida* species, causing candidemia, especially invasive candidiasis. *Candida albicans* is the primary pathogenic fungus in humans, causing serious, invasive infections.

In our work, investigation of the antimicrobial activity of synthesized AgNPs showed encouraging results (Figure 7 and Figure 8). Therefore, AgNPs synthesized by *Ganoderma lucidum* exhibited antimicrobial activity against all tested strains of Gram-positive and Gram-negative bacteria and yeast, in both tests, by using, respectively, 30 μL and 50 μL aliquot samples. It can be noted that the bacteria were more susceptible to AgNPs activity, compared to the fungi tested, the AgNPs acting more as antibacterial agents than as anticandidal agents. Our AgNPs were better against *Pseudomonas aeruginosa* (16.1 ± 0.2 mm diameter of inhibition zone), *S. aureus* and *C. parapsilosis* (equal value of 16.0 ± 0.1 mm), and *E. coli* (15.9 ± 0.1) mm. We considered that AgNPs can have beneficial effects on health and the environment, inhibiting the proliferation of these pathogens and reducing the risk to the environment and to public health.

Our results are consistent with the results of the antimicrobial activity of fungal-mediated green synthesis of AgNPs in the literature. Hence, AgNPs synthesized by other *Ganoderma* sp., namely *Ganoderma sessile* [48] and *Ganoderma applanatum* [30], demonstrated antimicrobial efficiency against *Escherichia coli, Staphylococcus aureus*, and *Pseudomonas aeruginosa*. As with our study, a higher antibacterial effect against *Staphylococcus aureus, Pseudomonas aeruginosa,* and *Escherichia coli* was reported for AgNPs synthesized by *Ganoderma lucidum*, as compared with the antifungal effect on *Candida albicans* [29,49]. In addition, there are reports on the antimicrobial effects of silver nanoparticles synthesized by other *Basidiomycetes*, such as *Polyporus plorans* [50], *Pleurotus ostreatus* [51], and *Pleurotus giganteus* [45].

## 5. Conclusions

The aqueous extracts of mycelia from *Ganoderma lucidum* contain bioactive compounds that may be useful in the synthesis of nanoparticles with antimicrobial activity. The biosynthesized AgNPs were investigated through different techniques, such as UV-Vis spectra, FTIR analysis, XRD, and SEM. The AgNPs synthesized by *Ganoderma lucidum* presented mainly antibacterial effects against several bacterial strains, such as *E. coli, P. aeruginosa,* and *S. aureus*. In light of the above, we consider that the biosynthesized AgNPs proved to have potential for health and environmental applications and for treatment of microbial infections. In addition to this, the use of edible and medicinal mushrooms is safe for human health, since *Ganoderma* has been widely used in traditional medicine.

Further studies will be focused on the optimization of biogenic microbial synthesis of silver nanoparticles, deepening on their characterization, and insisting on the achievement of the nanoparticles stabilization by using biocompatible materials without affecting antibacterial activity.

## Data Availability

Not applicable.
